# Associations between substandard housing and depression: insights from the Korea welfare panel study

**DOI:** 10.1186/s12888-020-03011-2

**Published:** 2021-01-07

**Authors:** Selin Kim, Wonjeong Jeong, Bich Na Jang, Eun-Cheol Park, Sung-In Jang

**Affiliations:** 1 Department of Public Health, Graduate School, Yonsei University, Seoul, Republic of Korea; 2 Institute of Health Services Research, Yonsei University, Seoul, Republic of Korea; 3 Department of Preventive Medicine & Institute of Health Services Research, Yonsei University College of Medicine, 50 Yonsei-ro, Seodaemun-gu, Seoul, 03722 Republic of Korea

**Keywords:** Minimum housing standard, Substandard housing, Depression

## Abstract

**Background:**

Housing is an important social determinant of health. Poor housing conditions are associated with a wide range of health conditions, including mental health. The study aimed to investigate the association between substandard housing and depression.

**Methods:**

We used panel data collected by the Korea Welfare Panel Study and a sample drawn from waves 11 (2016) to 13 (2018). Substandard housing was defined via three criteria: the minimum residential area and number of rooms by application, essential facility standards, and environmental standards. Depression was measured with the CESD-11. A generalized estimating equation model was used to investigate associations between substandard housing and CESD-11 scores.

**Results:**

Participants living in substandard housing have higher depression scores (male: β = 0.63, female: β = 0.40) than participants who do not live in substandard housing. Participants who do not meet environmental standards have higher depression scores (male: β = 0.85, female: β = 0.66) than participants who do not live in substandard housing; the findings are seen in both men and women.

**Conclusion:**

This study identified an association between substandard housing and depression by gender, and the results were significant. We found that among the three criteria, environmental standards are most likely to be associated with depression. In practical terms, we should consider improving environmental factors of housing to mitigate mental health issues related to substandard housing.

**Supplementary Information:**

The online version contains supplementary material available at 10.1186/s12888-020-03011-2.

## Background

The human right to adequate housing, which is derived from the right to an adequate standard of living, is of central importance for the enjoyment of all economic, social, and cultural rights [1]. Acknowledging this truth, in 1991, the UN committee on Economic, Social, and Culture Rights announced seven elements of adequate housing (legal security of tenure; availability of services, materials, facilities, and infrastructure; affordability; habitability; accessibility; location; and cultural adequacy) [2]. The committee emphasized that the right to housing should not be interpreted in a narrow or restrictive sense; rather, it should be seen as the inalienable right to live somewhere in security, peace, and dignity.

The Republic of Korea has established minimum housing standards and has been actively working to reduce the number of households living in substandard housing units. These minimum housing standards were first introduced in 2000 and later updated in 2011 [3]. As of 2000, about 3.3 million (23.4%) of Korea’s households did not meet the minimum standards [4]. The percentage of households falling beneath the minimum housing standard was significantly reduced to 7.2% in 2012 and 5.7% in 2018 [5]. This reduction could be explained by expansion of the public rental housing program [6] and massive scale of housing build up and redevelopment [7]. When examined according to income class, the lower the income level, the higher the ratio of households under the minimum housing standards [8].

The public health community has grown increasingly aware of the importance of the social determinants of health, including housing [9]. Poor housing conditions are associated with a wide range of health conditions, including respiratory infections, asthma, lead poisoning, injuries, and mental health issues [10]. Previous studies show that people living in problematic housing have a greater likelihood of experiencing poor mental health [11–13]. One study used 6 items of poor housing conditions (not enough light; lack of adequate heating; condensation; leaky roof; damp walls or roof etc.;) and 12-item General Health Questionnaire to analyzed the association between substandard housing and mental health [14]. Another study used 4 items of substandard housing quality (tapping the cleanliness of the home, the number of rooms in the home, the safety of the building’s interior, and safety of the area outside the building) and allostatic load to assessed the association between substandard housing and stress [15].

Similarly, some previous researches suggest that inadequate housing is associated with depression. One study shows that housing instability and disarray are associated with screening positive for depression [16]. Another study indicates that poor quality of housing is associated with depression initially and overtime [17]. However, these studies are restricted to children and females with children. Also, measurement of substandard housing and depression are based on self-report.

Therefore, the study aimed to investigate the potential association between substandard housing and depression using objective measurement tools.

## Methods

### Data and population

We used data from the Korea Welfare Panel Study (KoWePS) conducted by the Korea Institute for Health and Social Affairs and Seoul National University. KoWePS is an annual longitudinal panel survey that began in 2006. It includes 18,856 individuals from 7072 households who were recruited by a two-stage stratified cluster sampling at 2006. Interviews are conducted interviews using a computer assisted personal interviewing (CAPI) technique. This data is suitable for use in low-income policy or poverty studies because about 50% of the samples are low income earners with a median income of 60% or less. This study used a sample drawn from waves 11 (2016) to 13 (2018). We used data as cross-sectionally and the outcomes are at waves 11, 12 and 13 respectively. Among the population of 15,989 in 2016, we excluded 5585 individuals who provided no answer for survey questionnaires. Thus, the 2016 data included a total of 10,404 individuals.

### Measures

The primary outcome of interest in this study was depression. Depression was measured based on the 11-item version of the Center for Epidemiologic Studies Depression Scale (CESD-11). The CESD-11 is a shorter version of the original 20-item instrument and is a well-validated self-reported screening tool [18]. The CESD-11 comprises 11 questions; the total score is calculated by adding the scores for all questions and multiplying this value by 11. Scores range from 0 to 60, where higher scores indicate increased depressive symptoms.

Substandard housing was our key independent variable. In this study, the operationalization of substandard housing was based on both Korean minimum housing policy and previous research [19]. There are three detailed criteria for the minimum housing standard: the minimum residential area for each household composition and the number of rooms by application, the essential facility standards, and the construction and environmental standards (Supplementary 1). For minimum residential area, the standard criteria were applied to households comprising one to six people, and for households with seven to nine people, it was set by adding square meterage based on the standard. In other words, we cumulatively added 9m^2^—which is the difference between a five-person and a six-person household—to 55m^2^, which is the standard for six-person households. The number of rooms was based on one room for one to two people, two rooms for three people, three rooms for four to five people, four rooms for six to eight people, and five rooms for nine people. The minimum housing standards include provisions for essential facilities for daily life, such as access to clean water and sewer facilities, a single standing kitchen, single flush toilet, and single bath facilities. If any of these standards were not met, the location was regarded as substandard housing. Lastly, in relation to construction and environmental standards, KoWePS includes four indicators: structural material should be heat-resistant, fire-resistant, provide for heat dissipation, and be moisture-proof; provide appropriate soundproofing, ventilation, lighting, and heating facilities; comply with standards of noise, vibration, odor, and air pollution; and not be located in areas where there is a significant risk of natural disasters such as tidal waves, floods, mountain accidents, or cliff collapse. In this study, if two or more of the above four indicators were not met, the housing was considered to not meet environmental standards. Collectively, these three detailed criteria—the residential area and number of rooms, essential facilities, and environmental standards—are defined as comprising substandard housing.

Demographic, socioeconomic, and housing variables and health-related factors were included in the study. Demographic variables included gender, household type, age, and region. Socioeconomic variables included education level, marriage status, income level (quartile), and employment status. Housing variables included housing benefit, housing tenure, and housing type. Health-related factors included smoking status, alcohol consumption, perceived health status, and chronic diseases.

### Statistical analysis

We calculated the distribution of the general characteristics at baseline. Analysis of variance (ANOVA) was used to analyze mean CESD-11 scores at time points for categories of experiencing material deprivation. To analyze the effect of substandard housing on depression, we used the generalized estimating equation (GEE) model. The GEE model is known to be efficient and to provide unbiased regression estimates for use in analyzing longitudinal or repeated measures research designs with non-normal response variables [20]. Statistical analyses were performed using the GENMOD procedure in SAS version 9.4 (SAS Institute, Inc., Cary, NC, USA). The results were considered statistically significant if the *p*-value was less than 0.05.

## Results

Table 1 presents the general characteristics at baseline (2016). Of the 10,404 participants, 15.6% male and 15.3% female live in substandard housing. Participants who live in substandard housing have higher mean scores on the CESD-11 than participants who do not live in substandard housing (live in substandard housing males: 2.88; do not live in substandard housing males: 2.88; live in substandard housing females: 4.32; do not live in substandard housing females: 3.85).

**Table 1:** General characteristics of the sample at baseline (2016)

Variables	CESD-11											
	Male					***P*** value	Female					***P*** value
	N	%	MEANS	±	SD		N	%	MEANS	±	SD	
**Substandard housing**												
No	3,940	84.4	2.88	±	3.97	0.0013	4,856	84.7	3.85	±	4.46	0.0069
Yes	731	15.6	3.56	±	4.39		877	15.3	4.32	±	4.69	
**Household type**												
Without disabled members	4,189	89.7	2.82	±	3.93	0.3255	5,341	93.2	3.75	±	4.40	<.0001
With disabled members	482	10.3	4.36	±	4.71		392	6.8	6.24	±	5.13	
**Age (years)**												
< 30	507	10.9	2.09	±	3.56	0.0607	678	11.8	2.76	±	3.94	<.0001
30-39	692	14.8	2.20	±	3.56		756	13.2	2.47	±	3.72	
40-49	957	20.5	2.42	±	3.63		945	16.5	2.63	±	3.73	
50-59	794	17.0	2.81	±	3.97		897	15.6	3.60	±	4.26	
60-69	680	14.6	3.17	±	3.99		933	16.3	4.34	±	4.51	
≥ 70	1,041	22.3	4.46	±	4.58		1,524	26.6	5.88	±	4.91	
**Region**												
Metropolitan	1,977	42.3	2.83	±	3.99	0.1666	2,444	42.6	3.64	±	4.50	0.0235
Rural	2,694	57.7	3.09	±	4.08		3,289	57.4	4.12	±	4.50	
**Education level**												
Middle school or under	1,297	27.8	4.38	±	4.57	<.0001	2,600	45.4	5.26	±	4.82	0.0004
High school	1,543	33.0	2.69	±	3.80		1,468	25.6	3.04	±	4.05	
College or above	1,831	39.2	2.24	±	3.57		1,665	29.0	2.60	±	3.72	
**Marriage status**												
Living w/ spouse	4,480	95.9	3.03	±	4.07	0.0817	5,632	98.2	3.95	±	4.51	0.1511
Living w/o spouse	191	4.1	1.80	±	3.24		101	1.8	2.16	±	3.42	
**Income level**												
Low	947	20.3	4.68	±	4.70	0.0003	1,664	29.0	5.69	±	4.92	<.0001
Lower middle	1,255	26.9	3.23	±	4.06		1,416	24.7	3.80	±	4.40	
Upper middle	1,253	26.8	2.35	±	3.63		1,318	23.0	3.07	±	4.06	
High	1,216	26.0	2.06	±	3.39		1,335	23.3	2.67	±	3.71	
**Employment type**												
Permanent employee	2,503	53.6	2.49	±	3.64	0.0129	1,290	22.5	3.39	±	4.27	0.2980
Temporary employee	545	11.7	2.65	±	3.80		787	13.7	3.11	±	4.12	
Daily hired employee	359	7.7	3.59	±	4.19		844	14.7	4.18	±	4.34	
Nonemployee	1,264	27.1	3.93	±	4.63		2,812	49.0	4.31	±	4.70	
**Housing benefit**												
Yes	4,462	95.5	2.86	±	3.95	<.0001	5,420	94.5	3.83	±	4.45	0.5875
No	209	4.5	5.58	±	5.09		313	5.5	5.42	±	5.17	
**Housing Tenure**												
Owner-occupation	3,126	66.9	2.82	±	3.93	0.0099	3,617	63.1	3.69	±	4.36	<.0001
Rental	1,545	33.1	3.31	±	4.24		2,116	36.9	4.30	±	4.72	
**Housing Type**												
Detached dwelling	1,168	25.0	3.62	±	4.30	0.4465	1,541	26.9	4.74	±	4.67	0.5302
Multi-family house	3,321	71.1	2.77	±	3.94		3,966	69.2	3.61	±	4.40	
Commercial building	182	3.9	2.70	±	3.79		226	3.9	3.71	±	4.50	
**Smoking status**												
Current smoker	1,639	35.1	2.96	±	4.04	0.0420	120	2.1	5.91	±	5.29	0.0010
Non-smoker	3,032	64.9	3.00	±	4.05		5,613	97.9	3.88	±	4.47	
**Alcohol consumption**												
Yes	1,519	32.5	3.37	±	4.28	0.0623	3,885	67.8	4.16	±	4.60	0.0015
No	3,152	67.5	2.79	±	3.91		1,848	32.2	3.40	±	4.25	
**Percieved health status**												
Healthy	4,093	87.6	2.67	±	3.82	<.0001	4,785	83.5	3.49	±	4.27	<.0001
Unhealthy	578	12.4	5.21	±	4.83		948	16.5	6.09	±	5.01	
**Chronic diseases**												
Yes	2,406	51.5	2.25	±	3.48	<.0001	2,577	45.0	2.78	±	3.91	0.0042
No	2,265	48.5	3.76	±	4.43		3,156	55.0	4.85	±	4.73	
**Total**	**4,671**	**100.0**	**2.99**	**±**	**4.05**		**5,733**	**100.0**	**3.92**	**±**	**4.50**	

Table 2 reports the results of the GEE model analysis of factors associated with depression. Participants living in substandard housing demonstrate higher scores of depression (male: β = 0.63, female: β = 0.40) than participants who do not live in substandard housing. Disabled participants have higher depression scores (male: β = 0.43, female: β = 0.68) than participants without disabilities. Low-income participants tended to have higher depression scores (male: β = 0.84, female: β = 0.88) than people with high incomes. Participants who receive a housing benefit demonstrated higher depression scores (male: β = 0.85, female: β = 0.64) than participants who do not receive housing benefit assistance. Moreover, participants who rent their homes showed higher depression scores (male: β = 0.36, female: β = 0.35) than those living in owner-occupied homes.

**Table 2:** Results of the generalized estimating equation analysis of factors associated with depression

Variables	CESD-11					
	Male			Female		
	ß	S. E	***P*** value	ß	S. E	***P*** value
**Substandard housing**						
No	Ref.			Ref.		
Yes	0.63	0.11	<.0001	0.40	0.11	0.0002
**Household type**						
without disabled members	Ref.			Ref.		
with disabled members	0.43	0.15	0.0045	0.68	0.18	0.0001
**Age (years)**						
< 30	Ref.			Ref.		
30–39	0.26	0.15	0.0824	−0.16	0.14	0.2544
40–49	0.35	0.15	0.0169	−0.41	0.14	0.0040
50–59	0.44	0.16	0.0064	0.07	0.17	0.6772
60–69	0.20	0.17	0.2331	0.18	0.19	0.3465
≥ 70	0.81	0.19	<.0001	1.03	0.21	<.0001
**Region**						
Metropolitan	Ref.			Ref.		
Rural	0.14	0.08	0.0942	0.24	0.09	0.0047
**Education level**						
Middle school or under	0.55	0.14	<.0001	0.79	0.16	<.0001
High school	0.14	0.10	0.1722	0.19	0.11	0.0831
College or above	Ref.			Ref.		
**Marriage status**						
Living w/ spouse	Ref.			Ref.		
Living w/o spouse	−0.23	0.17	0.1611	−0.61	0.23	0.0071
**Income level**						
Low	0.84	0.15	<.0001	0.88	0.14	<.0001
Lower middle	0.24	0.11	0.0203	0.32	0.11	0.0024
Upper middle	0.08	0.09	0.3867	0.15	0.10	0.1247
High	Ref.			Ref.		
**Employment type**						
Permanent employee	Ref.			Ref.		
Temporary employee	0.14	0.12	0.2288	−0.19	0.12	0.1080
Daily hired employee	0.46	0.15	0.0024	−0.31	0.13	0.0146
Nonemployee	0.47	0.11	<.0001	−0.08	0.10	0.4283
**Housing benefit**						
Yes	0.85	0.23	0.0002	0.64	0.19	0.0009
No	Ref.			Ref.		
**Housing Tenure**						
Owner-occupation	Ref.			Ref.		
Rental	0.36	0.09	<.0001	0.35	0.09	<.0001
**Housing Type**						
Detached dwelling	Ref.			Ref.		
Multi-family house	−0.06	0.10	0.5786	0.15	0.11	0.1484
Commercial building	−0.17	0.19	0.3668	−0.03	0.20	0.8800
**Smoking status**						
Current smoker	0.37	0.08	<.0001	1.23	0.30	<.0001
Non-smoker	Ref.			Ref.		
**Alcohol consumption**						
Yes	0.11	0.08	0.1854	0.24	0.08	0.0026
No	Ref.			Ref.		
**Percieved health status**						
Healthy	Ref.			Ref.		
Unhealthy	1.19	0.13	<.0001	1.05	0.11	<.0001
**Chronic diseases**						
Yes	0.53	0.08	<.0001	0.46	0.09	<.0001
No	Ref.			Ref.		

Table 3 outlines the subgroup analysis for the association between substandard housing and depression by covariates. Participants with no disability or disabled household members who live in substandard housing have higher depression scores than non-disabled participants who live in standard housing (male: β = 0.62, female: β = 0.41). Moreover, participants who live with disabled household members in substandard housing have higher depression scores than participants who live with disabled household members in standard housing. However, this association was not statistically significant in either men or women. While male participants who’s housing tenure is rental and live in substandard housing have higher depression scores than owner-occupation (rental: β = 0.59, owner-occupation: β = 0.65), female participants who’s housing tenure is owner-occupation and living in substandard housing have higher depression scores than rental (rental: β = 0.30, owner-occupation: β = 0.52).

**Table 3:** Subgroup analysis of substandard housing association to depression stratified by covariates

Variables	CESD-11			
	No	Yes		
	ß	ß	S. E	***P*** value
**Male**				
**Household type**				
Without disabled members	Ref.	0.62	0.12	<.0001
With disabled members	Ref.	0.67	0.38	0.0784
**Housing Tenure**				
Owner-occupation	Ref.	0.59	0.17	0.0005
Rental	Ref.	0.65	0.15	<.0001
**Housing Type**				
Detached dwelling	Ref.	0.88	0.23	0.0002
Multi-family house	Ref.	0.56	0.14	<.0001
Commercial building	Ref.	0.22	0.41	0.6011
**Female**				
**Household type**				
Without disabled members	Ref.	0.41	0.11	0.0002
With disabled members	Ref.	0.23	0.43	0.5849
**Housing Tenure**				
Owner-occupation	Ref.	0.52	0.16	0.0015
Rental	Ref.	0.30	0.15	0.0368
**Housing Type**				
Detached dwelling	Ref.	0.35	0.21	0.0924
Multi-family house	Ref.	0.44	0.13	0.0009
Commercial building	Ref.	0.58	0.39	0.1358

Table 4 shows the results of association between substandard housing and depression based on the three criteria of substandard housing. Male participants who do not meet the minimum requirements for residential area and number of rooms have higher depression scores than those who do not live in substandard housing (β = 0.53). Participants who do not meet construction and environmental standards have higher depression scores than participants who do not live in substandard housing with regard to both men and women (male: β = 0.85, female: β = 0.66).

**Table 4:** Association between substandard housing and depression based on three criteria of substandard housing

Variables	CESD-11					
	Male			Female		
	ß	S.E	***P*** value	ß	S.E	***P*** value
**Substandard housing**						
No	Ref.			Ref.		
Yes						
Minimum residential area and number of rooms	0.53	0.13	<.0001	0.22	0.12	0.0712
Essential facility	0.52	0.33	0.1156	0.27	0.31	0.3724
Construction and environmental standards	0.85	0.19	<.0001	0.66	0.19	0.0006

## Discussion

In this study, we examined the potential association between substandard housing and depression. The main findings suggest that living in substandard housing is related to depression. Participants who live without disabled members in their household have association with depression when they live in substandard housing. Among the three criteria for substandard housing, living in a situation that fails to meet construction and environmental standards is most likely to be associated with depression.

Previous studies regarding substandard housing and mental health problems suggest similar associations. One study suggests that damp, moldy, and cold indoor conditions may be associated with anxiety and depression [21]. Another study indicates that living in neighborhoods where noise impedes sleep is associated with poor mental health [22]. Similarly, in our study indicates that participants who live in house that does not meet construction and environmental standards such as noise, vibration, odor, air pollution, ventilation have higher depression score. Further, living in substandard housing may be related to behavioral problems among children [23]. Similar results were found in this study that living in substandard housing that does not meet the minimum residential area and number of rooms are associated with mental health problem. Although the targeted population were different. Pragmatic concerns and fear may explain these results; living in substandard housing is a psychosocial stressor that can lead to mental health problems [10]. There is strong accumulated evidence that episodic stressors play a causal role in many instances of depression [24].

A key finding of this study was that the results of our subgroup analysis of substandard housing in association with depression stratified by household type. Regardless of whether the participant or a member of their household was disabled, if that participant lives in substandard housing, they are likely to have a higher depression score than a participant living in standard housing. However, the results were only statistically significant in participants without disabled household members. This result may be explained by the cash benefits offered by a disability pension, which is an income support for people with severe disabilities [25]. Moreover, in South Korea, disabled people benefit from the National Basic Livelihood Security System (NBLSS). The NBLSS is an integrated benefit system comprising four areas: Livelihood Benefit, Medical Benefit, Housing Benefit, and Education Benefit [26]. The housing benefit includes a discount to one’s electricity and heating bills. As economic burdens elevate the risk of depression [27], financial support may act as a buffer, lowering the risk of depression.

Another key finding was that among the three minimum housing standards criteria, construction and environmental standards are most likely to be associated with depression. Construction and Environmental standards include noise, vibration, odor, air pollution, ventilation, and so on. It is well known that prolonged exposure to damp, mold, noise, and odor in one’s living environment are associated with mental health issues [21, 22, 28, 29]. However, an interesting result of this study was that essential facilities, including water and sewage, a single standing kitchen, single flush toilet, and single bath facilities, do not appear to be associated with depression. One possible explanation for this result is that most participants may meet the essential facility criteria; however, further research is needed in this area.

Evaluations of housing renewal programs for people living in poor conditions, as well as research regarding early educational interventions, show that improving social conditions can contribute to improved mental health status [30]. Korea officially works to support people living in substandard housing via several programs [4]. The Public Rental Housing Programs comprise the main low-income housing policies in Korea. Those enrolled in these programs receive favorable treatments in terms of land acquisition, long-term financing below market rates, and tax deductions and exemptions. To assist with housing acquisition and expenses, there are two types of Demand-Side Programs: the deposit loan program and the housing benefits program. The deposit loan program lends some portion of the required deposit at a below-market interest rate, whereas the housing benefits program offers a cash subsidy support based on household size.

Despite these efforts, a UN report [31] revealed that in Korea, the number of people who live in substandard housing is increasing. This poses a serious threat to wellbeing: those who live in substandard housing are easily exposed to greater risks of fire and crime as well as the risks inherent to the poor living environment. The report also indicates that women, young people, the elderly, migrant workers, people with disabilities, and sexual minorities living in substandard housing are more often subject to discrimination and social exclusion from residential welfare.

This study has several limitations. First, measures of substandard housing are based on self-report; therefore, under- or overestimation of behaviors and conditions may have affected the accuracy of reporting among participants. Second, we could not include a factor related to the contextual appropriateness of participants’ housing cost burden, such as ability to pay their rent or mortgage, or being able to buy a home. However, we were able to include some housing-related variables such as housing benefit, housing tenure, and housing type. Third, because personality characteristics and medical history are likely to be associated with depression, our inability to include these in our statistical models may have resulted in exaggerating the associations under investigation.

Despite these limitations, this study has several strengths. First, this study worked to identify associations by using three-year longitudinal data relying on a national sample; thus, the results may be generalized to the Korean population as a whole with some confidence. The study expands our knowledge of the effects of substandard housing outside its most common context. Most previous studies in this area have targeted European populations, rarely studying the effects of substandard housing in Asian populations. Thus, this study contributes to the literature by using minimum housing standards as a measure of substandard housing and its potential association to depression among South Koreans.

## Conclusion

In conclusion, this study identified an association between substandard housing and depression by gender, and the results were significant. We found that among the three criteria for substandard housing, construction and environmental standards are most likely associated with depression. Despite the Korean government’s efforts to provide housing assistance of various types and to improve standards of living throughout the nation, some portion of the population still lives in substandard housing. Based on this study, we should consider means of improving housing environmental factors to mitigate the psychosocial stresses of living in substandard housing, thus helping to combat depression and improve mental health among this population.

## Supplementary Information

**Additional file 1.**

## Data Availability

https://www.koweps.re.kr:442/main.do;jsessionid=64D4F10703F4D419DF13D77E6104A44B

## Abbreviations

OR: Odds ratio
CI: Confidence interval
KoWePS: Korea Welfare Panel Study
CESD-11: 11-item version of the Center for Epidemiologic Studies Depression Scale
ANOVA: Analysis of variance
GEE: Generalized estimating equation
NBLSS: National Basic Livelihood Security System
