# Supplementary material for: Associations between substandard housing and depression: insights from the Korea welfare panel study
Source: BMC Psychiatry. 2021 Jan 7;21:12. doi: 10.1186/s12888-020-03011-2 (PMC7792032; doi:10.1186/s12888-020-03011-2)
Supplement: Supplementary file 1 — Additional file 1. [file 12888_2020_3011_MOESM1_ESM.docx]

| **Supplement 1. Three detailed criteria for the Korean minimum housing standard** | | | |
| --- | --- | --- | --- |
| Minimum residential area and  number of rooms | Minimum  residential area | 1~6 people | 55㎡ |
|  |  | 7~9 people | 64㎡ |
|  | Number  of rooms | 1~2 people | 1 room |
|  |  | 3 people | 2 rooms |
|  |  | 4~5 people | 3 rooms |
|  |  | 6~8 people | 4 rooms |
|  |  | 9 people | 5 rooms |
| Essential facility standards | | Single water and sewage, single standing kitchen, single flush toilet, single bath facilities | |
| Construction and  environmental standard | | Heat resistance; fire resistance; heat dissipation; moisture-proofing; soundproofing; ventilation; lighting; heating facilities; noise; vibration; odor; air pollution; natural disasters such as tidal waves, floods, mountain accidents, cliff collapse | |
|  |  |  |  |
